# DNA Methylation Profiling in IDH‐Mutant Gliomas: Biological Evolution, Molecular Grading and Clinical Implementation

**DOI:** 10.1111/nan.70100

**Published:** 2026-08-22

**Authors:** Pietro Tralongo, Gabriele Ricciardi, Mariagiovanna Ballato, Vincenzo Fiorentino, Valeria Zuccalà, Giorgia Castellani, Quintino Giorgio D'Alessandris, Guido Fadda, Lucia Ricci‐Vitiani, Maria Caffo, Maurizio Martini

**Affiliations:** ^1^ Department of Biomedical, Dental, Morphological and Functional Imaging Sciences University of Messina Messina Italy; ^2^ Department of Human Pathology of Adults and Developmental Age “Gaetano Barresi,” Division of Pathology University of Messina Messina Italy; ^3^ Department of Oncology and Molecular Medicine Istituto Superiore Di Sanità, Viale Regina Elena 299 Rome Italy; ^4^ Department of Neurosurgery Fondazione Policlinico Universitario a. Gemelli IRCCS, Largo a. Gemelli, 8 Rome Italy; ^5^ Department of Biomedical and Dental Sciences and Morphofunctional Imaging, Unit of Neurosurgery University of Messina Messina Italy

**Keywords:** DNA methylation, glioma CpG island methylator phenotype, IDH mutation, molecular classification, prognostic biomarkers

## Abstract

Mutations in isocitrate dehydrogenase 1 or 2 (*IDH1/2*) define a major subgroup of adult‐type diffuse gliomas and induce accumulation of D‐2‐hydroxyglutarate, resulting in extensive DNA and histone methylation changes. This epigenetic reprogramming underlies the glioma CpG island methylator phenotype (G‐CIMP) and contributes to tumour lineage, differentiation state and clinical behaviour. DNA methylation profiling has consequently evolved from a research tool into an important ancillary method for CNS tumour classification, molecular subclassification and copy‐number analysis. This narrative review examines the biological basis, analytical platforms and clinical applications of DNA methylation profiling in IDH‐mutant gliomas. Particular attention is given to the distinction between research‐defined G‐CIMP‐high and G‐CIMP‐low states and classifier‐defined lower‐grade and high‐grade methylation subclasses. High‐grade signatures, including A_IDH_HG and G‐CIMP‐low, are associated with adverse genomic features and outcome but should not be considered interchangeable or used in isolation to assign CNS WHO grade. Their interpretation requires integration with histology and established molecular criteria, including homozygous *CDKN2A/B* deletion. The review also discusses array‐based profiling, emerging Nanopore and long‐read approaches, computational quality control, and the integration of methylation data with genomic and transcriptomic alterations. Longitudinal studies indicate that a subset of IDH‐mutant astrocytomas undergoes methylation loss, copy‐number accumulation and altered cell‐state differentiation during progression, although these changes are not universal. The clinical relevance of *MGMT* promoter methylation is more context‐dependent than in IDH‐wildtype glioblastoma. Moreover, the INDIGO trial established the clinical activity of mutant IDH inhibition in selected Grade 2 gliomas but did not validate methylation class as a predictive biomarker or demonstrate complete epigenetic reversal. Emerging applications, including single‐cell and spatial epigenomics, tumour microenvironment deconvolution and CSF methylation profiling, remain investigational. Overall, DNA methylation profiling is most valuable when used as integrated diagnostic and risk‐stratification evidence within the complete histological, genomic and clinical context.

## Summary

- IDH1/2 mutations generate D‐2‐hydroxyglutarate and establish a broad hypermethylated G‐CIMP phenotype, but the methylome remains heterogeneous and can evolve during tumour progression.
- G‐CIMP‐low and classifier‐defined A_IDH_HG identify adverse biology, yet they are methodologically distinct and must not be used alone to assign CNS WHO Grade 4.
- Genome‐wide DNA methylation profiling can support tumour classification and molecular risk stratification while providing broad copy‐number information; array‐based platforms remain the most established approach.
- Clinical interpretation requires integration with histology and genomics, particularly established grading features such as homozygous CDKN2A/B deletion, while Nanopore, single‐cell, spatial and CSF methylation applications remain emerging or investigational.

## Graphical abstract

Mutant IDH1/2 produces D‐2‐hydroxyglutarate, establishing the G‐CIMP methylation phenotype while permitting epigenetic evolution during glioma progression. DNA methylation profiling supports tumour classification, lower‐ versus high‐grade subclassification and broad copy‐number assessment, but A_IDH_HG/G‐CIMP‐low signatures must be integrated with histology and established genomic grading criteria such as homozygous CDKN2A/B deletion. Array‐based profiling is currently the most established approach, whereas rapid Nanopore, single‐cell, spatial and CSF applications remain emerging.

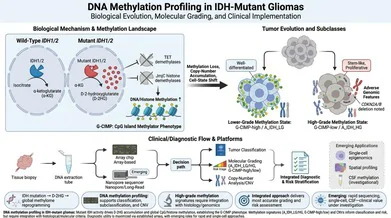

## 1 Introduction

Adult‐type diffuse gliomas are currently classified according to an integrated histomolecular framework in which mutations of isocitrate dehydrogenase 1 or 2 (*IDH1/2*) and whole‐arm 1p/19q codeletion define the principal tumour types [1]. IDH‐mutant diffuse gliomas therefore comprise astrocytoma, IDH‐mutant, and oligodendroglioma, IDH‐mutant and 1p/19q‐codeleted. Within these entities, histological features, genomic alterations and DNA methylation profiles provide complementary information regarding classification, grading and clinical behaviour.

Recurrent *IDH1* and *IDH2* mutations were identified as early, lineage‐defining events in diffuse gliomagenesis [2]. Mutant IDH enzymes acquire a neomorphic activity that converts α‐ketoglutarate into D‐2‐hydroxyglutarate (D‐2HG). Accumulation of D‐2HG inhibits α‐ketoglutarate‐dependent dioxygenases, including ten‐eleven translocation DNA demethylases and Jumonji C‐domain histone demethylases, producing widespread alterations of DNA and histone methylation. The identification of the glioma CpG island methylator phenotype (G‐CIMP), strongly associated with IDH mutation and favourable outcome, established the epigenome as a central component of IDH‐mutant glioma biology [3]. Experimental expression of mutant *IDH1* subsequently demonstrated that the mutation itself can initiate broad DNA hypermethylation, altered transcription and impaired differentiation [4].

The relevance of methylation profiling now extends beyond recognition of an IDH‐associated hypermethylated phenotype. Genome‐wide profiles can support CNS tumour classification, identify prognostically distinct subclasses and generate broad copy‐number information from the same assay. Integrated studies have also shown that IDH‐mutant astrocytomas and oligodendrogliomas retain distinct genomic and epigenetic identities despite sharing an initiating metabolic alteration [5].

Within astrocytoma, IDH‐mutant, current classifier frameworks distinguish lower‐grade and high‐grade methylation subclasses. These groups differ in copy‐number complexity and outcome and may refine risk beyond conventional morphology [6]. The classifier‐defined high‐grade state overlaps biologically with G‐CIMP‐low, but the two designations derive from different analytical procedures and should not be considered interchangeable.

Several limitations complicate clinical translation. Classifier outputs are probabilistic and depend on tissue quality, tumour purity, platform, preprocessing, reference cohort and classifier version. Formal G‐CIMP‐high or G‐CIMP‐low assignment is not a routine output of every clinical classifier. Moreover, many observations derive from small retrospective cohorts or have been extrapolated from IDH‐wildtype glioblastoma, which differs from IDH‐mutant glioma in genomic evolution, microenvironment and natural history.

The relationship between methylation subclass and molecular grading is particularly important. Homozygous *CDKN2A/B* deletion is an established criterion for CNS WHO Grade 4 astrocytoma, IDH‐mutant, even without necrosis or microvascular proliferation [1]. High‐grade methylation signatures, including A_IDH_HG and G‐CIMP‐low, identify clinically aggressive subsets but are not independent Grade 4 criteria. cIMPACT‐NOW Update 12 therefore recommends that they be integrated with histological, genomic and clinical findings [7].

This narrative review examines the biological basis, analytical technologies and clinical applications of DNA methylation profiling in IDH‐mutant gliomas. Particular attention is given to G‐CIMP states, classifier‐defined lower‐ and high‐grade subclasses, longitudinal and spatial evolution, multi‐omic integration, molecular grading and therapeutic implications. Evidence specific to IDH‐mutant gliomas is distinguished from pan‐glioma observations and exploratory applications.

## 2 Literature Search Strategy

A structured literature search was conducted using PubMed/MEDLINE, Scopus and Web of Science. Publications from January 2008, corresponding to the initial recognition of recurrent IDH mutations in diffuse glioma, through June 2026 were considered. Search terms included combinations of ‘IDH‐mutant glioma’, ‘IDH‐mutant astrocytoma’, ‘oligodendroglioma’, ‘DNA methylation’, ‘methylome’, ‘G‐CIMP’, ‘G‐CIMP‐high’, ‘G‐CIMP‐low’, ‘methylation classifier’, ‘methylation class high‐grade’, ‘glioma progression’, ‘intratumoral heterogeneity’, ‘DNA methylation array’, ‘Oxford Nanopore’, ‘long‐read sequencing’, ‘single‐cell methylation’, ‘spatial methylation’, ‘cerebrospinal fluid’, ‘liquid biopsy’ and ‘IDH inhibitor’.

Priority was given to original studies specifically investigating adult‐type IDH‐mutant gliomas, longitudinal or multiregional cohorts, large molecular‐classification studies, prospective clinical trials and consensus or classification documents. Studies of IDH‐wildtype glioblastoma were included only when they addressed general technological or analytical principles potentially transferable to IDH‐mutant tumours. Mixed cohorts without subgroup‐specific results were not used to support disease‐specific conclusions.

Reference lists of relevant original articles, consensus documents and authoritative reviews were screened for additional studies. Conference abstracts were considered only when no corresponding peer‐reviewed full article was available. Owing to heterogeneity in platforms, tumour populations, molecular endpoints and clinical outcomes, no formal risk‐of‐bias assessment or quantitative meta‐analysis was performed. Evidence was synthesised narratively, with emphasis on disease specificity, study design, independent replication and clinical maturity.

## 3 Biological Basis and Evolution of DNA Methylation in IDH‐Mutant Gliomas

### 3.1 IDH Mutations and the Glioma CpG Island Methylator Phenotype

Mutations involving *IDH1* or *IDH2* are early molecular events in most adult diffuse lower‐grade gliomas and define a biologically distinct tumour group [2]. The commonest alteration is *IDH1* p.R132H, whereas other substitutions at IDH1 R132 and IDH2 R172 are less frequent. Mutant enzymes reduce α‐ketoglutarate to D‐2HG, which accumulates at high concentrations and interferes with multiple α‐ketoglutarate‐dependent enzymes.

**FIGURE 1:**
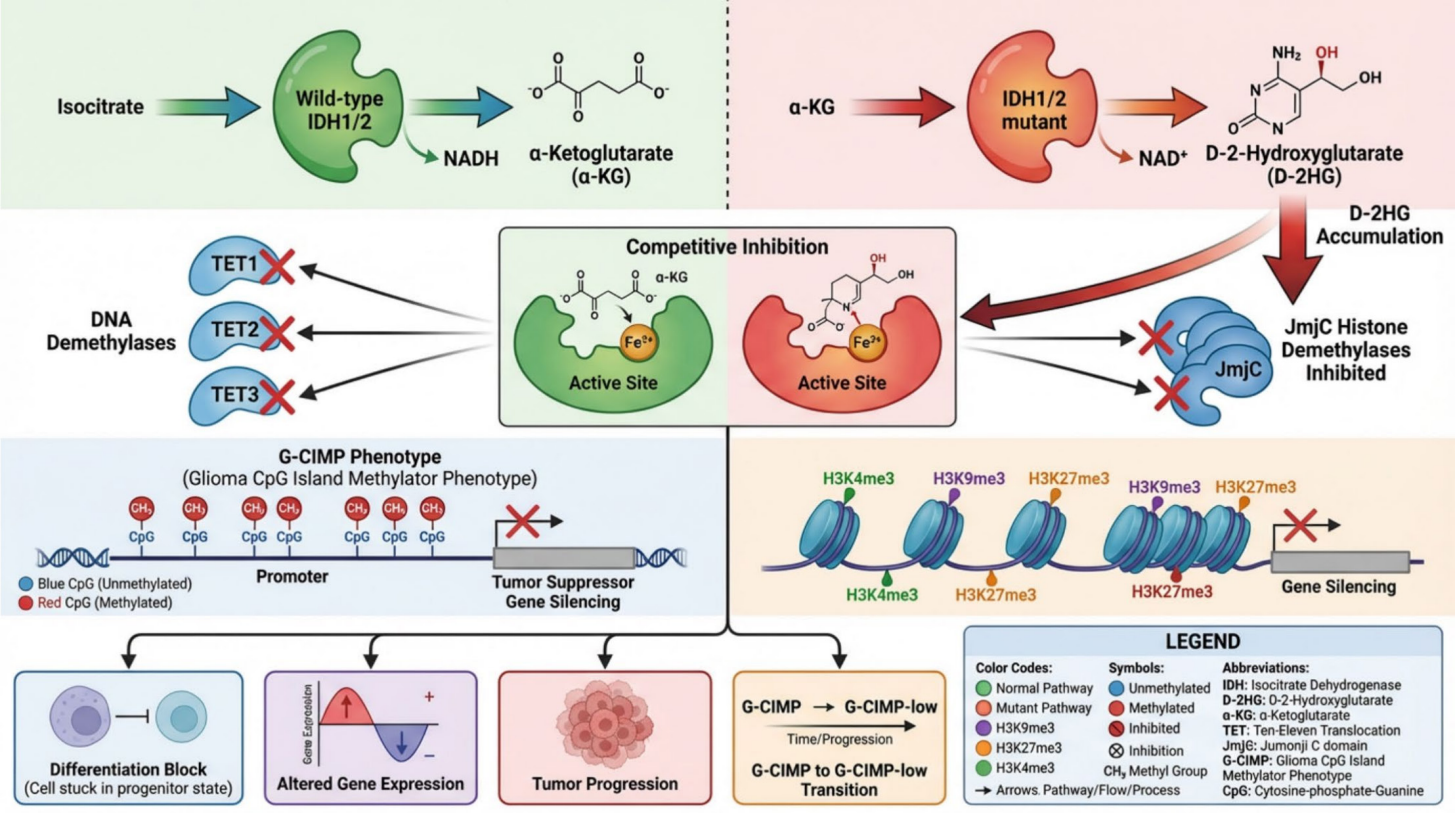
Biological consequences of mutant IDH activity and their relationship with methylome evolution in IDH‐mutant gliomas. Wild‐type IDH1 and IDH2 normally catalyse the conversion of isocitrate to α‐ketoglutarate (α‐KG). Mutant IDH1 or IDH2 acquires a neomorphic enzymatic activity that converts α‐KG into D‐2‐hydroxyglutarate (D‐2HG). Accumulation of D‐2HG competitively inhibits α‐KG‐dependent dioxygenases, including ten‐eleven translocation DNA demethylases and Jumonji C‐domain histone demethylases, resulting in widespread alterations of DNA and histone methylation. These changes contribute to the glioma CpG island methylator phenotype, impaired cellular differentiation and transcriptional reprogramming. During tumour progression, a subset of IDH‐mutant astrocytomas undergoes relative DNA methylation loss, accumulation of copy‐number alterations and transition toward more aggressive epigenetic and transcriptional states. Such evolution is neither universal nor determined solely by treatment exposure. Mutant IDH inhibition reduces D‐2HG production, but does not necessarily reverse the established methylation and chromatin landscape completely. Abbreviations: α‐KG, α‐ketoglutarate; D‐2HG, D‐2‐hydroxyglutarate; G‐CIMP, glioma CpG island methylator phenotype; IDH1/2, isocitrate dehydrogenase 1/2; JmjC, Jumonji C domain; TET, ten‐eleven translocation enzyme.

Inhibition of TET‐family demethylases contributes to accumulation of methylated cytosines across regulatory and non‐regulatory regions. In parallel, inhibition of Jumonji C‐domain histone demethylases alters chromatin organisation. These changes affect transcription, cellular differentiation and lineage commitment, generating an epigenetic state that favours gliomagenesis by restricting normal differentiation programmes (Figure 1) [4].

Noushmehr et al. described coordinated hypermethylation of numerous CpG islands and termed this state G‐CIMP [3]. G‐CIMP was associated with IDH mutation, younger age and improved survival compared with non‐G‐CIMP gliomas. Experimental introduction of mutant *IDH1* reproduced widespread methylation changes and impaired differentiation, supporting a causal relationship between mutant IDH activity and the phenotype [4].

Nevertheless, IDH mutation does not produce an identical methylome in every tumour. Lineage, cell of origin, cooperating alterations, anatomical context, microenvironment and disease stage influence the resulting profile. Astrocytomas and oligodendrogliomas consequently retain distinct methylation and transcriptional programmes [5].

IDH mutations also occur in other neoplasms, including central chondrosarcoma and chondroma [8] and intrahepatic cholangiocarcinoma [9]. However, their transcriptional and epigenetic consequences differ according to tissue context [10]. G‐CIMP should therefore be regarded as a broad biological framework rather than a single uniform state. Its definition has varied according to platform, selected CpGs, clustering strategy and threshold, complicating comparison among studies and translation into routine diagnostics [11].

### 3.2 Mutation‐Specific Epigenetic Effects

Different IDH variants may have unequal metabolic and epigenetic effects. In 1p/19q‐non‐codeleted astrocytomas, non‐R132H *IDH1/2* mutations have been associated with greater genome‐wide methylation and lower overall gene expression than *IDH1* p.R132H (cfr. Table 1) [12]. In the CATNON cohort, non‐R132H mutations were also associated with improved outcome, with a reported hazard ratio of 0.41 [12].

**TABLE 1:** Reported epigenetic and clinical differences between IDH1 p.R132H and non‐R132H IDH1/2 mutations in 1p/19q‐non‐codeleted astrocytomas.

Feature	IDH1 p.R132H	Non‐R132H IDH1/2 mutations	Interpretation and limitations
Relative frequency in adult diffuse glioma	Most common IDH alteration	Less frequent; heterogeneous IDH1 and IDH2 variants	Frequencies vary by tumour type, age and population
Relative D‐2HG production	Lower than several non‐R132H variants in experimental models	Higher for several variants	Variant‐specific enzymatic activity is not uniform across all non‐R132H mutations
Genome‐wide DNA methylation	Comparatively lower methylation in reported 1p/19q‐non‐codeleted astrocytoma cohorts	Comparatively higher methylation	Cohort‐level association; not sufficient for individual prognostication
Genome‐wide gene expression	Comparatively higher expression	Comparatively lower expression	Likely related partly to differences in methylation intensity
Assignment to adverse methylation subclasses	More frequent in CATNON‐associated analysis	Less frequent	May contribute to outcome differences but does not establish direct causality
Clinical outcome in 1p/19q‐non‐codeleted astrocytoma	Comparatively less favourable in CATNON population	Improved outcome compared with IDH1 p.R132H; reported HR 0.41, 95% CI 0.24–0.71	Interpret within selected CATNON cohort; do not generalise automatically to all IDH‐mutant gliomas
Independent grading relevance	None	None	IDH mutation subtype is not currently an independent CNS WHO grading criterion
Predictive relevance for IDH inhibitors	Not established	Not established	D‐2HG differences are biologically plausible but not prospectively validated for therapy selection
Current diagnostic reporting	Report exact IDH variant	Report exact IDH variant	Use standard HGVS nomenclature; avoid grouping all non‐R132H variants without acknowledging heterogeneity

Abbreviations: D‐2HG, D‐2‐hydroxyglutarate; HGVS, Human Genome Variation Society; IDH1/2, isocitrate dehydrogenase 1/2.

One possible explanation is that several non‐R132H mutants produce more D‐2HG than IDH1 p.R132H, causing stronger inhibition of α‐ketoglutarate‐dependent enzymes. However, lineage, co‐occurring alterations and patient selection may also contribute. These associations should therefore not be interpreted as evidence that mutation subtype alone determines prognosis.

Variant‐specific D‐2HG production could theoretically influence methylation intensity and response to IDH inhibition. At present, however, IDH variant subtype is neither an independent grading criterion nor a validated treatment‐selection biomarker.

### 3.3 Longitudinal Methylome Evolution

Although IDH mutation is generally retained, the associated methylome is not static. Comparisons of primary and recurrent IDH‐mutant gliomas have identified variable methylation loss, copy‐number accumulation and transcriptional reprogramming during progression [13, 14, 15, 16, 17].

A subset of recurrent tumours shifts from a highly methylated state toward G‐CIMP‐low‐like or classifier‐defined high‐grade profiles [13]. This transition has been associated with increased copy‐number burden, activation of proliferative or stem‐like programmes and poorer outcome. Its frequency, however, varies according to tumour lineage, platform, classification method, sampling time and cohort composition and should not be regarded as an inevitable pathway.

The biological effect of methylation loss depends on genomic distribution. Demethylation of enhancers, developmental elements and Polycomb‐associated regions may reactivate programmes linked to plasticity and malignant progression [14, 15]. Matched‐sample studies have documented changes in methylation subclass and progressive genomic alterations, particularly in astrocytoma [16]. More recent single‐nucleus analyses linked methylation loss with altered differentiation trajectories and expansion of stem‐like malignant‐cell states [17].

Treatment may shape these changes through selective pressure and evolutionary bottlenecks [14], but tumour‐intrinsic progression also contributes. Available studies cannot always distinguish direct treatment‐induced methylation changes from expansion of pre‐existing or newly emerging subclones. Treatment history, interval to recurrence, histological progression, tumour purity and genomic evolution should therefore be considered when interpreting paired samples.

Repeat methylation profiling may document retention or change of class and reveal newly acquired copy‐number events in selected recurrences. It is not currently required in every recurrent glioma, and apparent class shifts should not be interpreted independently of morphology and genomics.

### 3.4 Spatial Heterogeneity and Stability of Classification

Multiregional analyses show regional variability in locus‐specific methylation, copy‐number alterations and subclonal genomic events [18, 19]. A single tissue block may therefore fail to capture every molecular feature. However, broad methylation‐class identity is often more stable than individual CpG or copy‐number abnormalities.

Mapping of IDH‐mutant astrocytomas demonstrated regional divergence while retaining an overarching IDH‐mutant epigenetic identity [18]. A larger image‐guided study similarly found substantial concordance of broad methylation classification despite regional differences in tumour purity and individual features [19].

Single‐cell multimodal studies further demonstrate populations with distinct genetic, transcriptional and epigenetic states [20]. These states differ in differentiation, proliferation and stress response, although mixed IDH‐mutant and IDH‐wildtype cohorts limit disease‐specific interpretation.

Spatial heterogeneity has practical implications. Areas with low cellularity, treatment effect or abundant non‐neoplastic components may generate low‐confidence results. Histological review should therefore guide selection of viable and representative tumour. Profiling multiple regions may be informative in selected heterogeneous cases but is not a routine clinical requirement.

## 4 Technologies for DNA Methylation Profiling

### 4.1 Array‐Based DNA Methylation Profiling

Illumina Infinium arrays remain the most extensively validated platforms for CNS tumour methylation classification. The HumanMethylation450 BeadChip interrogates approximately 450,000 CpGs [21], whereas EPIC and EPIC v2 interrogate approximately 850,000–935,000 sites, with increased representation of regulatory regions [22, 23].

Although arrays cover only a fraction of the human methylome, their probes are enriched for informative CpG islands, promoters, gene bodies and enhancers. Their clinical value derives from reproducible measurement of a feature set suitable for supervised classification. Probe intensities can also generate broad copy‐number profiles, allowing tumour classification and chromosomal assessment from one assay [24, 25].

EPIC v2 redistributed and expanded probe content relative to preceding arrays [22, 23]. Cross‐platform studies require harmonisation because classifiers trained on earlier arrays may rely on probes absent from newer versions. The same limitation applies to longitudinal comparisons and prognostic signatures based on platform‐specific features.

Advantages of arrays include standardisation, compatibility with FFPE‐derived DNA, established analytical pipelines and large reference cohorts [24, 25]. Limitations include fixed probe content, incomplete representation of repetitive and intergenic regions, bisulfite‐associated DNA degradation and inability to detect most variants, balanced rearrangements or gene fusions directly.

Whole‐genome bisulfite sequencing offers broader, near‐single‐base coverage but is costly and computationally demanding. Reduced‐representation bisulfite sequencing enriches CpG‐dense regions at lower cost but provides incomplete, non‐uniform coverage and is less suited to broad copy‐number analysis or established classifiers. Both therefore remain principally research tools. The main technologies used for DNA methylation profiling in glioma, together with their advantages, limitations and current clinical maturity, are summarised in Table 2.

**TABLE 2:** Current and emerging technologies for DNA methylation profiling in glioma.

Technology	Principal role	Approximate coverage	Main advantages	Main limitations	Clinical maturity
Illumina 450K array	Historical platform for methylation‐based classification and research profiling	~450,000 targeted CpG sites	Large legacy datasets; compatible with early classifier cohorts; copy‐number inference from intensity data	Discontinued; lower regulatory coverage than EPIC; fixed probe content; bisulfite conversion	Historically established; mainly archival/research relevance
Illumina EPIC/EPIC v2 arrays	Current array‐based tumour classification, subclassification and broad copy‐number profiling	~850,000–935,000 targeted CpG sites	Most clinically mature; FFPE compatible; established pipelines and reference cohorts; methylation plus CNV output	Selected CpG coverage only; platform differences affect comparisons; no direct detection of most variants/fusions	Clinically mature in selected diagnostic settings
Whole‐genome bisulfite sequencing	Comprehensive methylome research and discovery	Potentially most genomic CpGs, depth‐dependent	Broad single‐base coverage; regulatory/intergenic/repetitive regions accessible	Costly; computationally demanding; bisulfite degradation; limited routine diagnostic integration	Research‐level
Reduced‐representation bisulfite sequencing	Focused analysis of CpG‐rich regions	CpG islands/promoters/enriched regions	Lower sequencing burden than WGBS; high resolution in selected regions	Incomplete and non‐uniform coverage; less suitable for CNV and established classifiers	Research‐level
Oxford Nanopore/long‐read methylation‐aware sequencing	Rapid or intraoperative classification; simultaneous methylation, CNV and selected sequence information	Potentially genome‐wide, effective coverage depth‐dependent	Direct native‐DNA methylation detection; real‐time analysis; no bisulfite conversion; long reads	Variable yield; DNA‐quality dependent; algorithm/calibration/regulatory standardisation still evolving	Emerging; prospectively validated but not universally available
Targeted or sparse methylation sequencing	Rapid classification using selected informative regions	Restricted or sparse genome‐wide coverage	Reduced time/input; compatible with rapid workflows	Performance depends on feature selection and reference representation; limited discovery potential	Emerging
Single‐cell/single‐nucleus methylome or chromatin profiling	Research of cell‐state heterogeneity and plasticity	Sparse single‐cell coverage	Separates malignant and non‐malignant populations; resolves subclonal states	High cost; sparse data; complex integration; small cohorts	Research‐level
Spatial epigenomic profiling	Mapping molecular states within anatomical niches	Method‐dependent	Links epigenetic state to histology, invasion and microenvironment	Technically heterogeneous; limited standardisation; few IDH‐mutant‐specific datasets	Research‐level
CSF/cfDNA methylation profiling	Minimally invasive classification or monitoring	Variable and often low tumour‐derived cfDNA	Potential option when tissue is inaccessible; possible serial monitoring	Low concentration; variable shedding; incomplete classifier validation in adult IDH‐mutant gliomas	Investigational

Abbreviations: CNV, copy‐number variation; CSF, cerebrospinal fluid; FFPE, formalin‐fixed paraffin‐embedded; WGBS, whole‐genome bisulfite sequencing.

### 4.2 Long‐Read and Nanopore‐Based Profiling

Nanopore sequencing detects methylation directly in native DNA while simultaneously generating sequence and copy‐number information. Because data are produced continuously, classification can begin before the run is complete.

Low‐pass Nanopore sequencing has supported classification across multiple CNS tumour types [26]. Application to FFPE specimens has also produced usable methylation and copy‐number profiles [27], although fixation‐related fragmentation may reduce read length and yield.

The Sturgeon deep‐learning classifier used sparse Nanopore data for ultra‐rapid intraoperative classification and was prospectively tested during neurosurgical procedures [28]. Such analysis may complement frozen‐section or smear interpretation when tumour identity could influence surgical strategy but it should not be considered a morphological replacement.

Rapid‐CNS^2^ was subsequently validated in a multicentre cohort and provided early class and chromosome‐arm information followed by more complete molecular results with continued sequencing [29]. The same study introduced MNP‐Flex, a platform‐agnostic classifier covering 184 classes and applicable across arrays and several sequencing approaches [29].

Nanopore profiling is therefore moving toward clinical implementation but still faces variability in DNA quality, sequencing yield, methylation‐calling algorithms, classifier calibration and infrastructure. Moreover, successful pan‐CNS classification does not prove that sparse sequencing reproduces every prognostic G‐CIMP or A_IDH signature developed on arrays.

### 4.3 Preprocessing, Quality Control and Computational Analysis

Reliable classification requires robust laboratory and computational quality control. Pre‐analytical variables include fixation, block age, DNA extraction, fragmentation, tumour cellularity, necrosis and contamination by non‐neoplastic tissue.

Array preprocessing generally includes background correction, colour adjustment, probe filtering and normalisation. The minfi package provides a widely used framework [30], while noob and functional normalisation address background and technical variation [31, 32]. ENmix offers an alternative approach [33]. Comparative studies indicate that optimal preprocessing depends on platform, tissue type and analytical endpoint [34].

Problematic probes include those with poor signal, cross‐reactive sequences, nearby polymorphisms or inconsistent performance. Copy‐number loss may also distort methylation measurements; SeSAMe was developed to reduce artefactual calls caused by genomic deletions [35].

Batch effects related to laboratory, reagent lot, processing date, scanner or array generation are particularly relevant in retrospective and longitudinal studies. Correction should be applied cautiously because excessive adjustment may remove genuine biological differences.

Classifier interpretation must include the model and version, confidence score, quality metrics, tumour purity, reference‐class representation and concordance with morphology and genomics. A low‐confidence result is not a negative methylation test. It may indicate poor DNA, low tumour content, an under‐represented entity or genuine biological divergence. No confidence threshold is universally applicable across platforms.

Copy‐number plots also require expert review. Chromosome‐arm events are usually more robust than small focal changes, especially at low purity. Equivocal findings with grading consequences, particularly homozygous *CDKN2A/B* deletion, should be confirmed orthogonally.

Advanced computational models, including biologically organised neural networks, may improve classification and interpretation [36]. Clinical use nevertheless requires independent validation, cross‐platform stability and transparent management of low‐confidence and out‐of‐distribution samples.

## 5 DNA Methylation‐Based Classification and Molecular Grading

### 5.1 Available CNS Tumour Methylation Classifiers

Methylation classifiers compare an individual profile with reference tumour classes and provide probabilistic evidence of biological similarity. Their output must be integrated with morphology, immunohistochemistry and genomics [24, 37].

The Heidelberg classifier established the most widely adopted framework. Its original random‐forest model included a broad spectrum of adult and paediatric tumours and has confirmed, refined or revised diagnoses in clinically relevant subsets [24, 25]. Its prominence reflects historical priority, reference‐cohort size, class diversity and continuous updating rather than proven intrinsic superiority over every alternative method.

The expanded classifier includes 184 subclasses based on 7495 reference profiles [37]. Because new entities and subdivisions are added, reports should state the version used. Changes in output after reanalysis may represent taxonomic refinement rather than biological evolution.

Independent classifiers demonstrate that methylation classification is not restricted to one platform. A Northwestern workflow showed high concordance with the Heidelberg system after clinical laboratory validation [38]. Other institutional and paediatric frameworks differ in reference cohorts, preprocessing, nomenclature and scoring and should not be assumed to be directly equivalent.

Online availability of a research classifier does not establish regulatory authorisation. Laboratories must validate tissue selection, DNA processing, classification, thresholds and reporting [39]. Low‐confidence cases should undergo integrated reassessment rather than forced assignment to the highest‐scoring class.

### 5.2 Lower‐Grade and High‐Grade Methylation Classes

Astrocytoma, IDH‐mutant, can be divided into A_IDH_LG and A_IDH_HG subclasses [6, 37]. A_IDH_HG is enriched for increased copy‐number burden and cell‐cycle abnormalities and is associated with poorer survival [6]. Histological grade and methylation class are correlated but incompletely concordant: some Grade 2–3 tumours fall into A_IDH_HG, whereas some morphologically high‐grade tumours retain a lower‐grade profile.

These findings do not justify replacing CNS WHO grade with methylation subclass. Necrosis, microvascular proliferation and homozygous *CDKN2A/B* deletion remain established Grade 4 criteria [1]. A_IDH_HG should instead prompt careful reassessment for additional adverse histological or genomic features.

cIMPACT‐NOW Update 12 considers A_IDH_HG and G‐CIMP‐low signatures indicative of aggressive Grade 3–4 behaviour when interpreted with other findings [7]. Neither is an isolated criterion for Grade 4. Evidence is strongest in astrocytoma and should not be transferred directly to oligodendroglioma.

### 5.3 G‐CIMP‐High and G‐CIMP‐Low

G‐CIMP‐high tumours retain extensive hypermethylation, whereas G‐CIMP‐low tumours show relative methylation loss and more aggressive biological features [3, 5, 11, 13]. G‐CIMP‐high/low and A_IDH_LG/HG are related but distinct frameworks. G‐CIMP groups derive largely from unsupervised research clustering, whereas A_IDH subclasses are supervised classifier assignments [6, 24, 37].

The two high‐risk states overlap in hypomethylation, genomic instability and proliferative programmes, but their terms should not be used interchangeably. Reports should specify platform, algorithm and reference cohort. Formal G‐CIMP assignment often requires additional bioinformatic analysis and is not a universal clinical output.

The adverse significance of G‐CIMP‐low is supported by cross‐sectional and longitudinal studies [13, 14, 15, 16], but the frequency of transition varies and may be influenced by lineage, treatment, sampling and tumour purity. Progression toward G‐CIMP‐low is neither universal nor the only evolutionary route.

Clinical and scientific reports should therefore separate the integrated WHO diagnosis, classifier‐derived methylation class, research‐derived G‐CIMP status and genomic grading alterations.

### 5.4 Integrated Interpretation

A methylation match supporting astrocytoma should be interpreted with confirmation of *IDH1/2* mutation, ATRX and TP53 findings and absence of whole‐arm 1p/19q codeletion [1, 39]. An oligodendroglial methylation match does not independently establish oligodendroglioma, which requires an IDH mutation and whole‐arm codeletion.

Discordant findings should trigger reassessment of tissue quality, sampling, treatment effect, morphology, genomic results and reference‐class representation. The highest score is not equivalent to diagnostic truth. cIMPACT‐NOW Update 9 recommends integrated interpretation and validated laboratory procedures [39].

An optimal report should include tumour type and grade, platform and classifier version, assigned class or subclass, confidence metric, relevant copy‐number findings, concordance with defining genomic alterations and technical limitations. A high‐grade signature without a recognised Grade 4 criterion should be described as an adverse molecular feature rather than translated automatically into Grade 4 [7].

## 6 Integration of DNA Methylation With Genomic and Transcriptomic Alterations

### 6.1 Complementary Molecular Information

Methylation, genomic and transcriptomic analyses provide related but non‐interchangeable information. Methylation reflects lineage and epigenetic state; sequencing detects nucleotide and structural alterations; transcriptomics measures expression and fusion transcripts.

Array signals can infer chromosome‐arm changes, aneuploidy and selected focal alterations [24, 25]. However, arrays do not directly detect most variants, indels, balanced rearrangements or fusions. Machine‐learning models can predict selected genomic features from methylation data [40], but clinically consequential alterations require direct validated testing. Tumour purity, subclonality and event size affect copy‐number interpretation.

### 6.2 Genomic Risk Stratification in Astrocytoma

Astrocytoma, IDH‐mutant, commonly harbours ATRX and TP53 alterations [1, 5]. These support astrocytic lineage but do not independently determine grade. During progression, additional abnormalities affect cell‐cycle, receptor tyrosine kinase and PI3K signalling.

Homozygous *CDKN2A/B* deletion is the best‐established genomic grading marker and is sufficient for CNS WHO Grade 4 [1]. It must be distinguished from hemizygous loss or other pathway abnormalities. Its adverse effect is well supported, although not every A_IDH_HG tumour carries the deletion and some intact tumours behave aggressively [6, 41].

Amplifications of *CDK4*, *CDK6* or *CCND2* and alterations of *RB1* represent alternative mechanisms of RB‐pathway disruption. Copy‐number changes involving *CDKN2A*, *CDK4* and *PDGFRA* stratify risk among lower‐grade astrocytomas [42]. Alterations of *RB1*, *PIK3CA* and *PIK3R1* have also been associated with adverse behaviour [43].

cIMPACT‐NOW Update 12 associates *PDGFRA* amplification with highly aggressive, Grade 4‐like behaviour, whereas several RB/PI3K‐pathway and receptor alterations are considered more consistent with intermediate risk [7]. These recommendations provide interpretive guidance but do not make every event an independent WHO criterion.

*MYCN* amplification identifies a rare particularly aggressive subset [44], while extensive aneuploidy or high copy‐number burden may also indicate poorer outcome [45]. Neither currently has a universal threshold for assigning grade. Key genomic and epigenomic alterations are summarised in Table 3.

**TABLE 3:** Selected genomic and epigenomic alterations with diagnostic, prognostic or grading relevance in IDH‐mutant gliomas.

Alteration/output	Main tumour context	Diagnostic/grading/prognostic relevance	Interpretive cautions
IDH1/2 mutation	Adult‐type diffuse glioma	Defining alteration for astrocytoma, IDH‐mutant, and oligodendroglioma, IDH‐mutant and 1p/19q‐codeleted	IDH status must be established directly; methylation prediction is supportive, not definitive
Whole‐arm 1p/19q codeletion	Oligodendroglioma	Required diagnostic criterion with IDH mutation	Must distinguish whole‐arm codeletion from partial loss
ATRX loss/TP53 alteration	Astrocytoma, IDH‐mutant	Supports astrocytic lineage	Not independent grading criteria
Homozygous CDKN2A/B deletion	Astrocytoma, IDH‐mutant	Established CNS WHO grade 4 criterion even without necrosis or microvascular proliferation	Distinguish from hemizygous loss; confirm equivocal methylation‐derived calls orthogonally
A_IDH_HG methylation subclass	Astrocytoma, IDH‐mutant	Adverse methylation subclass associated with poorer outcome and genomic complexity	Not an isolated WHO Grade 4 criterion; interpret with histology and genomics
G‐CIMP‐low	Mostly astrocytoma, IDH‐mutant research cohorts	Adverse biological state with relative methylation loss	Research‐derived; not interchangeable with A_IDH_HG unless method specified
PDGFRA amplification	Astrocytoma, IDH‐mutant	Associated with highly aggressive behaviour; cIMPACT‐NOW Update 12: grade 4‐like risk	Not equivalent to a universally adopted WHO standalone criterion
CDK4/CDK6/CCND2 amplification; RB1 alteration	Astrocytoma, IDH‐mutant	RB‐pathway disruption; adverse/intermediate‐risk features	Support risk stratification; do not automatically assign Grade 4
MYCN amplification	Rare IDH‐mutant astrocytoma subset	Associated with particularly poor outcome in limited cohorts	Rare; not a standalone grading criterion
High total copy‐number burden/aneuploidy	Astrocytoma, IDH‐mutant	Associated with aggressive behaviour and A_IDH_HG enrichment	No universal threshold for grading
TERT promoter mutation	Oligodendroglioma background	Common supportive alteration in oligodendroglioma	Not individually required for diagnosis; not a grading criterion
CIC/FUBP1 alterations	Oligodendroglioma	Common lineage‐associated alterations; possible prognostic associations	Independent prognostic value inconsistent; not grading criteria
MGMT promoter methylation	Diffuse gliomas	Context‐dependent; strongest predictive validation in IDH‐wildtype glioblastoma	Frequent in IDH‐mutant gliomas due to hypermethylated phenotype; interpret cautiously

Abbreviations: CNV, copy‐number variation; CNS, central nervous system; G‐CIMP, glioma CpG island methylator phenotype; WHO, World Health Organisation.

### 6.3 Relationship Between Genomic Alterations and Methylation Subclass

A_IDH_HG tumours commonly show more aneuploidy, focal amplification and cell‐cycle alteration than A_IDH_LG tumours [6, 41]. Epigenetic and genomic progression may therefore occur in parallel: methylation loss may facilitate activation of developmental or proliferative programmes, while copy‐number changes promote clonal expansion [13, 14, 15, 16, 17].

Galbraith et al. showed that methylation subclass, aneuploidy and *CDKN2A/B* deletion provide overlapping but non‐equivalent prognostic information [41]. Thus, an A_IDH_HG, *CDKN2A/B*‐intact tumour may represent increased risk but should not automatically be graded 4. Conversely, necrosis, microvascular proliferation or unequivocal homozygous deletion establishes Grade 4 even if the methylation profile appears lower grade.

### 6.4 Genomic Context of Oligodendroglioma

Oligodendroglioma requires *IDH1/2* mutation and whole‐arm 1p/19q codeletion [1]. Partial losses are not equivalent. TERT promoter, *CIC* and *FUBP1* alterations are common but not individually required [5]. ATRX loss or diffuse strong p53 expression should prompt reassessment.

Inactivating *CIC* mutations have been associated with poorer outcome in some 1p/19q‐codeleted gliomas [46], but the independent prognostic contribution of *CIC*, *FUBP1* and related events remains inconsistent. cIMPACT‐NOW Update 12 did not identify additional genomic or epigenomic criteria sufficiently validated to refine oligodendroglioma grading [7]. Recurrences may acquire aneuploidy or hypermutation, but no universally accepted methylation‐based progression pathway exists.

### 6.5 Transcriptomic and Multi‐Omic Integration

Transcriptomic analysis can reveal the functional consequences of methylation at promoters, enhancers and developmental elements. Longitudinal multi‐omic studies associate progression with methylation loss, developmental reactivation, stem‐like states and adverse genomic events [14, 15, 16, 17].

Methylation signatures can predict selected genomic or transcriptional states [40], but indirect inference cannot replace direct measurement when diagnostic or therapeutic consequences are involved. Metabolic profiling has demonstrated associations between metabolic and epigenetic phenotypes [47]. Integrated methylome, transcriptome and metabolome analysis subsequently identified a clinically aggressive IDH‐mutant subgroup [48]. Such metabologenomic groups remain research‐level and are not established WHO entities or validated predictors of therapy.

## 7 Clinical Diagnostic Implementation

### 7.1 Position in the Integrated Workflow

DNA methylation profiling is an ancillary molecular tool, not a replacement for neuropathological assessment [1, 39]. Its principal value lies in resolving difficult cases, refining classification and providing broad copy‐number information [24, 25, 49].

Initial evaluation should establish morphology and IDH status. IDH1 p.R132H immunohistochemistry is an appropriate first‐line test in most adult supratentorial diffuse gliomas, followed by sequencing when negative but suspicion persists [1]. Once an IDH mutation is confirmed, astrocytoma and oligodendroglioma are distinguished through whole‐arm 1p/19q assessment, supported by ATRX and p53 findings.

Grade should be assigned using established histological and molecular criteria. A_IDH_HG or G‐CIMP‐low can provide additional risk information but are not independent Grade 4 criteria [7]. Profiling should therefore address a defined diagnostic or prognostic question rather than be applied automatically to every unequivocal tumour.

### 7.2 Indications

Relevant indications include limited or distorted tissue, unusual morphology, discordant immunohistochemical and genomic findings, uncertain lineage, unexpectedly aggressive features in an otherwise lower‐grade astrocytoma, marked transformation at recurrence, uncertainty regarding tumour identity and a need for broad copy‐number information [24, 25, 39, 49].

Methylation profiling may preserve tissue when several sequential assays would otherwise be required. However, broad output cannot compensate for inadequate sampling. Testing is less informative when defining molecular criteria already establish a coherent diagnosis and the result would not influence classification or management. The suspected entity must also be represented adequately within the classifier reference set [37, 39].

### 7.3 Tissue Selection and Pre‐Analytical Requirements

Histological review should identify viable representative tumour and minimise necrosis, haemorrhage, gliosis, inflammation and treatment effect [39, 49]. Macrodissection can increase purity but may reduce yield or select a limited subclone.

Increasing non‐neoplastic admixture reduces classifier confidence and copy‐number amplitude [50], although no universal tumour‐percentage threshold applies. FFPE material is generally suitable, but fixation, block age and fragmentation influence performance. Laboratories should validate combined criteria for DNA input, quality and tumour fraction [39, 50].

The primary specimen is preferable when the question concerns original classification; recurrence tissue may be preferable when transformation or acquired risk is under investigation. Paired analyses must account for differences in processing, tumour purity, treatment, platform and classifier version [12, 13, 14, 15, 16, 17, 39].

### 7.4 Analytical Quality Control and Interpretation

Quality control should include signal intensity, bisulfite‐conversion performance, probe metrics, sample identity, estimated tumour content and copy‐number quality [34, 35, 39, 49]. Classifier name and version must be documented.

Results below validated confidence thresholds should be described as low‐confidence or non‐classifiable, not methylation negative. The highest‐scoring class should be evaluated for biological plausibility, morphological and immunophenotypic concordance, defining genomic alterations, copy‐number support and possible alternative matches.

Poor DNA, low cellularity, non‐neoplastic contamination, incomplete reference representation or an evolved recurrent tumour may all produce low‐confidence results [39, 49, 50]. When possible, another representative block or an alternative assay is preferable to repeated analysis of the same inadequate material.

### 7.5 Interpretation of Copy‐Number Profiles

Methylation‐derived copy‐number analysis may support whole‐arm 1p/19q codeletion, homozygous *CDKN2A/B* deletion, focal amplification and overall chromosomal instability [39, 49]. Broad chromosome‐arm events are more robust than small or subclonal abnormalities [49, 50].

Whole‐arm codeletion must be distinguished from partial loss. Ambiguous results require confirmation. Homozygous *CDKN2A/B* deletion also requires caution because it may independently establish Grade 4. A clear deletion may be reported in a validated workflow, whereas an equivocal low‐amplitude event should be confirmed orthogonally. Other amplifications should be reported according to their evidence and not automatically translated into grade [7].

### 7.6 Integration Into the Final Report

The report should distinguish the integrated WHO diagnosis from methylation‐derived supporting information [1, 39]. It should include tumour type and grade, defining alterations, platform, classifier version, class or subclass, confidence score, relevant copy‐number findings, concordance and limitations.

A high‐grade methylation match without an established Grade 4 criterion should be described as an adverse risk feature. Discordance should be stated explicitly and possible technical or biological explanations discussed. ‘Not classifiable’ is preferable to ‘methylation negative’.

### 7.7 Role at Recurrence

Repeat profiling may be useful when recurrence shows marked morphological transformation, an unusually long interval, uncertain identity or a need to assess genomic evolution [12, 13, 14, 15, 16, 17]. It cannot independently distinguish viable tumour from treatment effect. Apparent class changes must be supported by adequate quality and concordant histological or genomic evidence. Routine repeat analysis of every recurrence is not justified.

### 7.8 Established and Emerging Clinical Outputs

Clinically mature outputs include selected tumour‐class assignment, broad copy‐number profiling and support for integrated diagnosis [24, 25, 39, 49]. Context‐dependent outputs include lower‐ versus high‐grade subclassification, methylation‐based *MGMT* assessment and longitudinal comparison. Formal G‐CIMP assignment, microenvironment deconvolution, single‐cell or spatial analysis, liquid biopsy, indirect mutation prediction and treatment selection from methylation alone remain investigational. These features are summarised in Table 4.

**TABLE 4:** Clinical maturity and interpretive requirements of outputs derived from DNA methylation profiling in IDH‐mutant gliomas.

Methylation‐derived output	Clinical maturity	Potential value	Minimum interpretive requirements
Tumour methylation‐class assignment	Clinically established in selected diagnostic settings	Supports diagnosis in difficult, limited or discordant cases	Classifier name/version; confidence score; tissue quality; morphology and genomic concordance
Broad copy‐number profile	Clinically useful adjunct	Identifies arm‐level events, amplifications, deletions and genomic complexity	Assess tumour purity; confirm equivocal grade‐defining focal events
A_IDH_LG/A_IDH_HG subclassification	Clinically relevant risk stratification, especially in astrocytoma	Refines prognosis beyond morphology in selected cases	Do not convert A_IDH_HG alone into CNS WHO Grade 4
Formal G‐CIMP‐high/G‐CIMP‐low assignment	Research or specialised analysis	Biological insight into hypermethylated versus methylation‐loss states	Specify platform, CpG set, algorithm and reference cohort
MGMT promoter methylation from array data	Context‐dependent	May inform alkylating‐agent context; useful when validated locally	Specify model/assay; interpret differently from IDH‐wildtype glioblastoma
Repeat methylation profiling at recurrence	Selected clinical/research use	Assesses class stability, acquired CNV and transformation	Compare same/compatible platforms; consider purity, treatment and classifier version
Microenvironment deconvolution	Investigational	Estimates immune/stromal components from bulk methylation	Computational estimate only; requires reference methylomes; not treatment‐selective
Single‐cell or spatial epigenomics	Investigational	Defines cell states, niches, plasticity and heterogeneity	High cost, sparse data, complex analysis; no routine reporting framework
CSF/cfDNA methylation profiling	Investigational	Potential tissue‐sparing or serial monitoring tool	Low tumour‐derived DNA; negative result cannot exclude tumour; limited adult IDH‐mutant validation
AI‐based integrated prediction	Investigational to emerging	Integrates methylation with genomic, imaging and clinical variables	External validation, calibration, interpretability and clinical‐utility evidence required

Abbreviations: CNV, copy‐number variation; CSF, cerebrospinal fluid; FFPE, formalin‐fixed paraffin‐embedded; G‐CIMP, glioma CpG island methylator phenotype; MGMT, O6‐methylguanine‐DNA methyltransferase.

## 8 Prognostic and Therapeutic Implications

### 8.1 Prognostic Significance

Methylation subclass, G‐CIMP state, copy‐number burden and selected genomic alterations provide complementary prognostic information. In astrocytoma, A_IDH_HG is associated with poorer outcome than A_IDH_LG [6], only partly explained by grade or homozygous *CDKN2A/B* deletion [41]. G‐CIMP‐low similarly identifies relative hypomethylation and adverse biology [5, 11, 13].

Longitudinal progression may involve class transition, copy‐number accumulation and expansion of stem‐like states [13, 14, 15, 16, 17], although recurrence profiles are influenced by treatment, sampling and cellular composition. Most methylation signatures are therefore best regarded as prognostic or risk‐stratifying rather than treatment‐predictive.

### 8.2 *MGMT* Promoter Methylation

The predictive role of *MGMT* methylation is strongest in IDH‐wildtype glioblastoma. In IDH‐mutant gliomas it is frequent as part of the broader hypermethylated phenotype and is confounded by lineage, grade and 1p/19q status.

The NOA‐04 biomarker analysis showed that the prognostic or predictive significance of *MGMT* depends on IDH status [51]. In IDH‐mutant tumours, it was more closely associated with the favourable molecular background than with an independent treatment interaction. Further analysis demonstrated non‐uniform prognostic value across IDH‐mutant subtypes [52].

Array data can estimate *MGMT* status through models such as MGMT‐STP27 [53], but performance depends on genetic and epigenetic context and values near the threshold [54]. Reports should therefore specify the method and avoid implying universal prediction of temozolomide benefit.

### 8.3 Biological Rationale for Mutant IDH Inhibition

Mutant IDH is a rational therapeutic target because it generates D‐2HG. Inhibition reduces the oncometabolite and may relieve suppression of α‐ketoglutarate‐dependent enzymes. However, an established methylome may persist because tumour cells acquire secondary genomic and epigenetic alterations. IDH inhibition should not be conceptualised as a complete epigenetic reset.

Perioperative vorasidenib and ivosidenib substantially reduced intratumoral D‐2HG in non‐enhancing IDH1‐mutant glioma [55]. Vorasidenib showed greater brain penetration and target engagement and induced changes in differentiation‐related programmes, but complete methylome normalisation was not demonstrated. A pilot perioperative safusidenib study also found target engagement and multi‐omic changes, although its small design did not establish clinical efficacy or the causal role of methylation reversal [56].

### 8.4 The INDIGO Trial

INDIGO was a randomised phase 3 trial of vorasidenib in patients with residual or recurrent Grade 2 IDH‐mutant glioma previously treated with surgery alone [57]. Participants had non‐enhancing or minimally enhancing disease and were suitable for observation rather than immediate radiotherapy or chemotherapy.

Vorasidenib significantly prolonged imaging‐based progression‐free survival and delayed the next therapeutic intervention [57]. The ability to postpone radiochemotherapy is relevant in younger patients with slowly growing tumours and long expected survival.

The results should not be generalised to Grade 3–4, rapidly progressive, substantially enhancing or heavily pretreated disease. INDIGO did not compare vorasidenib directly with standard radiotherapy or chemotherapy, and an overall‐survival benefit has not yet been established. The trial validated mutant IDH as a therapeutic target, not G‐CIMP or methylation subclass as predictive biomarkers.

### 8.5 Methylation State as a Candidate Response Biomarker

Highly methylated, genomically simpler tumours might remain more dependent on mutant IDH than evolved high‐grade tumours, but this hypothesis is unproven. Neither G‐CIMP status nor A_IDH subclass currently determines eligibility for IDH inhibitors. Treatment‐related methylation changes are also not validated surrogate endpoints. Prospective trials should evaluate baseline methylation, copy‐number burden and co‐alterations for genuine interactions with treatment rather than simple prognostic associations.

## 9 Emerging and Research Applications

### 9.1 Tumour Microenvironment Deconvolution

Bulk methylation profiles contain signals from malignant cells, immune cells, microglia, vascular cells and reactive glia. Deconvolution of more than 4000 glial and glioneuronal tumours associated estimated immune composition with methylation class and genomic alterations [58]. However, these estimates depend on reference methylomes and cannot replace direct cellular assessment, especially for overlapping populations such as microglia and macrophages.

### 9.2 Single‐Cell and Single‐Nucleus Epigenomics

Single‐cell approaches resolve heterogeneity obscured in bulk analysis. Single‐nucleus chromatin‐accessibility profiling demonstrated regulatory diversity in IDH1‐mutant gliomas [59]. Multimodal single‐cell analysis linked epigenetic heterogeneity with plasticity and environmental stress [20].

These methods can define malignant and non‐neoplastic populations and investigate lineage progression or resistance. However, sparse coverage, small cohorts, high cost, complex preprocessing and loss of fragile cellular populations limit routine use.

### 9.3 Spatial Epigenomics

Spatial approaches link molecular states with anatomical niches such as invasive margins, proliferative regions, hypoxia or treatment effect. Existing multiregional data suggest that local methylation and genomic heterogeneity can coexist with stable broad class identity [18, 19].

Spatial platforms differ in whether they measure DNA methylation, chromatin accessibility, histone marks or expression. Many require specialised tissue handling and remain poorly standardised. Demonstrating a high‐risk spatial niche does not yet prove that sampling or targeting it improves outcome.

### 9.4 Cerebrospinal‐Fluid Methylation Profiling

CSF cfDNA offers a potential minimally invasive method for CNS tumour classification and monitoring. Tumour‐derived DNA is often more abundant in CSF than plasma, but yield depends on tumour burden, location, grade and proximity to CSF spaces.

Methylation profiling of paediatric CSF cfDNA has demonstrated proof of principle for tumour classification [60]. These findings cannot be considered direct validation in adult IDH‐mutant gliomas, which may have lower cellular turnover and variable shedding. Low DNA concentration, fragmentation and incomplete representation of classifier CpGs remain important limitations. A negative CSF result cannot exclude tumour.

### 9.5 Monitoring Tumour Evolution

Serial profiling of tissue may reveal retention or change of class, methylation loss and newly acquired copy‐number abnormalities [13, 14, 15, 16, 17]. However, platform, purity and sampling differences may mimic biological evolution. No methylation‐derived measure is validated as a surrogate endpoint for survival or as a routine marker of treatment response.

### 9.6 Epigenetic Effects on Tumour Immunity

IDH‐associated metabolism may influence chemokine expression, antigen presentation and innate immune signalling. Experimental inhibition of mutant IDH1 activated double‐stranded DNA sensing and enhanced antitumour immunity [61]. The interaction between ATRX loss and mutant IDH1 also influenced innate immune responses in experimental glioma models [62].

These findings provide a rationale for combination strategies but remain predominantly preclinical. Neither immune deconvolution nor a methylation‐defined immune state has been prospectively validated to select immunotherapy.

### 9.7 Artificial Intelligence and Integrated Models

Machine‐learning models can combine methylation with genomics, imaging and clinical variables. They may identify interactions that are not apparent from individual biomarkers but are vulnerable to overfitting, batch effects and limited generalisability. External validation, calibration and transparent handling of missing or out‐of‐distribution data are essential. Clinical adoption requires evidence that the model improves diagnosis or management, not merely retrospective statistical performance.

### 9.8 Requirements for Clinical Translation

Emerging applications must demonstrate analytical validity, clinical validity and clinical utility. Studies should use predefined endpoints, independent cohorts, harmonised processing and comparison with current standards. Prognostic models must add value beyond age, tumour type, grade, resection and established genomics. Predictive biomarkers must demonstrate a treatment interaction. Cost, tissue requirements, infrastructure and turnaround time must also be considered.

## 10 Limitations of DNA Methylation Profiling in IDH‐Mutant Gliomas

### 10.1 Pre‐Analytical Limitations

Low tumour content, necrosis, haemorrhage, inflammation, reactive gliosis and treatment effect reduce tumour‐derived signal [39, 49, 50]. FFPE‐related fragmentation, block age and extraction influence arrays and Nanopore sequencing [27, 39, 49]. DNA quantity alone does not guarantee suitability.

Sampling bias is unavoidable because a single block may not represent all subclonal or regional abnormalities [18, 19]. Macrodissection can improve purity but may reduce yield or overselect one region.

### 10.2 Analytical and Platform‐Related Limitations

Arrays interrogate selected CpGs rather than the complete methylome. Differences among 450K, EPIC and EPIC v2 complicate cross‐platform and longitudinal comparisons [21, 22, 23]. Bisulfite conversion, background correction, filtering, normalisation and batch adjustment may alter results [30, 31, 32, 33, 34, 35].

Nanopore avoids bisulfite conversion but introduces variable yield, incomplete rapid coverage and dependence on platform‐specific algorithms [26, 27, 28, 29]. Array‐ and sequencing‐derived results are not automatically interchangeable. Broad copy‐number changes are generally more reliable than focal events, particularly in low‐purity samples.

### 10.3 Classifier‐Related Limitations

Classifiers are reference dependent and cannot reliably identify absent or under‐represented entities [24, 37, 39]. Scores reflect similarity rather than diagnostic truth. Thresholds differ among models, and version updates can alter class nomenclature or specificity.

Discordance with morphology or genomics may reflect an incorrect original diagnosis, poor tissue, treatment effect, biological divergence or classifier limitations [25, 39, 49]. Defining genomic criteria retains priority in adult‐type diffuse gliomas.

### 10.4 Limitations of G‐CIMP and High‐Grade Subclassification

G‐CIMP definitions vary by platform, feature set and clustering method [5, 11]. Formal assignment is not universally available, and G‐CIMP‐low is related to but not identical with A_IDH_HG [5, 6, 37]. Reported transition rates differ across longitudinal studies [13, 14, 15, 16, 17].

High‐grade signatures are associated with adverse outcome but are not independent WHO Grade 4 criteria [1, 7]. Their evidence is also considerably stronger in astrocytoma than in oligodendroglioma.

### 10.5 Limitations of Prognostic and Predictive Evidence

Most studies are retrospective and include heterogeneous diagnoses, treatments and endpoints. Historical categories do not map perfectly onto current WHO entities. Several genomic and multi‐omic associations require independent validation [42, 43, 44, 45, 46, 47, 48].

A prognostic association does not establish treatment prediction. This distinction is particularly relevant to *MGMT* methylation [51, 52, 53, 54] and methylation changes after IDH inhibition [55, 56, 57].

### 10.6 Extrapolation From IDH‐Wildtype Glioblastoma

IDH‐wildtype glioblastoma differs from IDH‐mutant glioma in initiating drivers, copy‐number architecture, immune composition and natural history. General technological principles may transfer, but biological conclusions regarding methylation evolution, immune response or treatment resistance require IDH‐mutant‐specific evidence. Mixed single‐cell cohorts should therefore not be presented as exclusively representative of IDH‐mutant disease [20].

### 10.7 Limitations of Emerging Applications

Deconvolution, single‐cell analysis, spatial profiling and CSF methylation remain research applications [58, 59, 60]. Deconvolution produces estimates rather than direct cell counts; single‐cell assays have sparse coverage; spatial methods are heterogeneous; and CSF analysis is limited by low tumour‐derived DNA.

AI models may perform well in retrospective cohorts but require external prospective validation [36, 40]. Experimental immune effects of IDH inhibition do not establish clinical efficacy or a validated immunotherapy biomarker [61, 62].

### 10.8 Access, Standardisation and Clinical Utility

Methylation profiling is not uniformly available. Implementation requires laboratory infrastructure, bioinformatics, validated classifiers, quality assurance and experienced interpretation [39, 49]. Access to an online research classifier is not equivalent to authorisation for clinical reporting.

Cost‐effectiveness has not been demonstrated in every setting. Profiling may reduce sequential testing in difficult cases but add limited value to unequivocal tumours. Conventional arrays may also have prolonged turnaround because of batching, while rapid Nanopore approaches require specialised validated infrastructure [28, 29].

### 10.9 Limitations of the Present Review

This is a narrative review without formal risk‐of‐bias assessment or meta‐analysis. Included studies differ in tumour classification, platform, treatment and clinical endpoint, limiting direct comparison. The field is evolving rapidly, particularly regarding classifier versions, rapid sequencing, molecular grading and IDH‐targeted therapy. The review focuses on adult‐type IDH‐mutant gliomas; studies of other CNS tumours were included only for transferable technological or analytical principles.

## 11 Conclusions

DNA methylation profiling is an important ancillary component of integrated CNS tumour diagnostics. In IDH‐mutant gliomas, it can support classification, refine subclassification and provide broad copy‐number information from one assay [24, 25, 39, 49]. Its greatest value lies in selected cases in which morphology and standard molecular findings are limited, discordant or atypical rather than in mandatory testing of every unequivocal tumour.

The IDH‐associated methylation phenotype arises from D‐2HG‐mediated epigenetic remodelling but is influenced by lineage, mutation subtype, secondary genomic alterations and progression [3, 4, 5, 12, 13, 14, 15, 16, 17]. A_IDH_HG and G‐CIMP‐low identify adverse biology, yet remain methodologically distinct and cannot independently establish CNS WHO Grade 4 [1, 6, 7, 41]. Established histological criteria and homozygous *CDKN2A/B* deletion retain grading priority; other genomic abnormalities provide differing levels of supportive risk information [7, 41, 42, 43, 44, 45].

Methylation profiling is less established as a predictive biomarker. The meaning of *MGMT* promoter methylation is context dependent in IDH‐mutant tumours [51, 52, 53, 54]. Similarly, INDIGO demonstrated the clinical activity of vorasidenib in selected Grade 2 IDH‐mutant gliomas but did not validate methylation subclass as a treatment‐selection marker [57]. D‐2HG suppression may alter transcription and differentiation without fully reversing the established methylome [55, 56].

Nanopore‐based classification, single‐cell and spatial epigenomics, microenvironment deconvolution and CSF profiling may expand future applications [26, 27, 28, 29, 58, 59, 60]. Most require prospective validation, standardisation and demonstration of clinical utility.

Overall, DNA methylation profiling should be interpreted as integrated diagnostic and biological evidence rather than an autonomous endpoint. Its clinical value depends on representative tissue, validated analytical workflows, classifier‐specific quality control and reconciliation with histological, genomic and clinical findings.

## Author Contributions

Pietro Tralongo, Maria Caffo and Maurizio Martini conceived of the topic, drafted the manuscript and edited the manuscript. Gabriele Ricciardi and Mariagiovanna Ballato designed figures and edited the manuscript. Lucia Ricci‐Vitiani procured funding for the research team and edited the manuscript. Vincenzo Fiorentino, Valeria Zuccalà, Giorgia Castellani, Quintino Giorgio D'Alessandris and Guido Fadda edited the manuscript.

## Funding

This work was supported by Associazione Italiana per la Ricerca sul Cancro, AIRC (IG2021 n.26515) to LRV. This work has been partially conducted under the National Plan for Complementary Investments to the PNRR, M6/C2, project ‘Biobanking, Biomarkers and Big Data: B3D for Personalized Treatment of Glioblastoma’ (project code: PNRR‐TR1‐2023‐12377972), funded by the Italian Ministry of Health to PT.

## Ethics Statement

The authors have nothing to report.

## Consent

The authors have nothing to report.

## Conflicts of Interest

The authors declare no conflicts of interest.

## Data Availability

Data sharing not applicable to this article as no datasets were generated or analysed during the current study.
